# The association of TNF-α −308G/A and −238G/A polymorphisms with type 2 diabetes mellitus: a meta-analysis

**DOI:** 10.1042/BSR20191301

**Published:** 2019-12-20

**Authors:** Xiaoliang Guo, Chenxi Li, Jiawei Wu, Qingbu Mei, Chang Liu, Wenjing Sun, Lidan Xu, Songbin Fu

**Affiliations:** 1 Laboratory of Medical Genetics, Harbin Medical University, 157 Baojian Road, Nangang District, Harbin 150081, China; 2 Key Laboratory of Preservation of Human Genetic Resources and Disease Control in China (Harbin Medical University), Ministry of Education, China

**Keywords:** -238G/A, -308G/A, meta analysis, single nucleotide polymorphisms, T2DM, TNF-α

## Abstract

Tumor necrosis factor-α (TNF-α) is involved in insulin resistance and has long been a candidate gene implicated in type 2 diabetes mellitus (T2DM), however the association between *TNF-α* polymorphisms -308G/A and -238G/A and T2DM remains controversial. The present study sought to verify associations between these polymorphisms and T2DM susceptibility using a meta-analysis approach. A total of 49 case–control studies were selected up to October 2018. Statistical analyses were performed by STATA 15.0 software. The odds ratios (ORs) and 95% confidence intervals were calculated to estimate associations. Meta-analyses revealed significant associations between *TNF-α* −308G/A and T2DM in the allele model (*P*=0.000); the dominant model (*P*=0.000); the recessive model (*P*=0.001); the overdominant model (*P*=0.008) and the codominant model (*P*=0.000). Subgroup analyses also showed associations in the allele model (*P*=0.006); the dominant model (*P*=0.004) and the overdominant model (*P*=0.005) in the Caucasian and in the allele model (*P*=0.007); the dominant model (*P*=0.014); the recessive model (*P*=0.000) and the codominant model (*P*=0.000) in the Asian. There were no associations between *TNF-α* −238G/A and T2DM in the overall and subgroup populations. Meta-regression, sensitivity analysis and publication bias analysis confirmed that results and data were statistically robust. Our meta-analysis suggests that *TNF-α* −308G/A is a risk factor for T2DM in Caucasian and Asian populations. It also indicates that *TNF-α* −238G/A may not be a risk factor for T2DM. More comprehensive studies will be required to confirm these associations.

## Introduction

Diabetes is a global epidemic, with an estimated worldwide prevalence of 1 in 11 adults (approximately 425 million people in 2017), and is projected to increase to 629 million people by 2045 (http://www.diabetesatlas.org/). Individuals with type 2 diabetes mellitus (T2DM) accounted for 90% of this total [1]. T2DM is a complex metabolic disorder and usually involves pancreatic islet dysfunction and insulin-secreting β cell failure in the endocrine pancreas (Islets of Langerhans), allowing for the secretion of more insulin to counteract insulin resistance in peripheral tissues (adipose, skeletal muscle and liver). Ultimately, T2DM shows an uncontrolled increase in blood glucose levels [2], therefore the pathogenesis of T2DM is insulin resistance [3].

Some *in vivo* and *in vitro* studies have shown that tumor necrosis factor-α (TNF-α) induces insulin resistance to some extent, through the inhibition of intracellular signaling from the insulin receptor [4,5]. The disease has a strong genetic component, however few genes have been identified [1]. Several genome-wide association scans (GWAS) have been performed for T2DM and several candidate genes have been proposed [6–10]. Of multiple candidate genes, the *TNF-α* promoter polymorphisms −308G/A and −238G/A have been studied in T2DM etiology [11].

Currently, it is inconclusive whether these polymorphisms (−308G/A and −238G/A) in the *TNF-α* promoter lead to T2DM susceptibility. Two large-scale British association analyses found these polymorphisms were not robustly associated with T2DM [11,12] and similar results have been observed in China [13,14] and India [15]. However, studies have also suggested that −308G/A and −238G/A are risk factors for T2DM in Egypt [16] and Iran [17]. Studies from different racial backgrounds may produce conflicting results and these independent studies are confusing and controversial. Therefore, we performed a large-scale meta-analysis to investigate associations between these polymorphisms and T2DM.

## Materials and methods

### Literature search

This meta-analysis was conducted according to the recommendations of the Preferred Reporting Items for Systematic Reviews and Meta-Analysis 2009 (PRISMA2009). All published studies up to October 2018 were searched using the PubMed, Embase, EBSCO, OVID, and Web of science database. We used the following terms: ‘TNF-α’, ‘TNF-alpha’, ‘tumor necrosis factor-α’, ‘tumor necrosis factor-alpha’, ‘T2DM’, ‘type 2 diabetes mellitus’, ‘type 2 diabetes’, ‘type II diabetes’, ‘non-insulin dependent diabetes’, ‘NIDDM’, ‘polymorphism’, ‘variation’, ‘−308G/A’, ‘rs1800629’, ‘−238G/A’ and ‘rs361525’. Relevant references in selected articles were also included.

All articles were independently reviewed by two investigators. Studies were assessed against the following inclusion criteria: (1) the associated study of *TNF-α* polymorphisms (−308G/A and −238G/A) with the risk of T2DM, (2) the study was case–control designed, (3) sufficient information on genotype frequencies (GG, AA and GA) in both cases and controls to estimate an odds ratio (OR) with a 95% confidence interval (95% CI), (4) all data were original. Exclusion criteria were as follows: (1) other DM (diabetes) types were excluded, (2) non-human studies, (3) reviews, meta-analysis and non-case–control studies and (4) studies not published in English.

### Quality score assessment

Study quality was assessed to guarantee the strength of results and conclusions. Quality assessment was performed according to the Newcastle–Ottawa Quality Assessment Scale (NOS), which is a validated scale for nonrandomized studies in meta-analyses [18]. This NOS uses a star system to assess the quality of a study in three domains: selection, comparability and outcome/exposure. The NOS assigns a maximum of 5 stars for selection (in the case of cross-sectional studies), 2 stars for comparability, and 3 stars for outcome/exposure. Studies achieving a score of at least 8 stars were classified as being at low risk of bias (i.e., thus reflecting the highest quality). A maximum of 9 scores, including selection, comparability and exposure items were awarded. Any score disagreements were decided by a third researcher.

### Data extraction

Data were independently extracted by two investigators using a standardized form. For each study, the following information was extracted: (1) name of first author; (2) year of publication; (3) ethnicity of population; (4) sample sizes and genotype distributions; (5) allele frequency of the major variant. Ethnicity was categorized as Caucasian, Asian and African.

### Statistical analysis

The Hardy–Weinberg equilibrium (HWE) test was calculated using the Chi-squared test. The distribution of allele frequencies in controls was considered to deviate from HWE when *P*<0.05. STATA (15.0; Stata Corporation, College Station, TX, U.S.A.) software was used to calculate meta-analysis results. Individual study heterogeneity was assessed by Cochran’s Q test and the *I^2^* statistic (*P*<0.10 and *I^2^* > 50% indicates evidence of heterogeneity) [19]. The fixed-effects model (Mantel–Haenszel method) was used to estimate the pooled OR [20], when there was no evidence of heterogeneity, otherwise the random-effects model (DerSimonian and Laird method) was used [20,21]. ORs with corresponding 95% CIs were calculated to assess associations between *TNF-α* promoter polymorphisms (−308G/A and −238G/A) and T2DM risks. Five genetic models were used in this meta-analysis: (1) the allele model (A allele vs. G allele); (2) the dominant model (GA+AA vs. GG); (3) the recessive model (AA vs. GA+GG); (4) the codominant model (GA vs. GG; AA vs.GG) and (5) the overdominant model (GG+AA vs. GA). A *P*-value <0.05 was accepted as the significant threshold for each genetic model. Three subgroups, including Caucasian, Asian and African, based on ethnicity, were analyzed to reduce influences from genetic backgrounds. A meta-regression was used to search the source of heterogeneity [22], which contained publication year, sample size, ethnicity, HWE and number of studies. The 10000 times Monte Carlo permutation test approach was used for assessing the statistical significance of meta-regression [23,24]. *I^2^ res* explained the proportion of residual variation due to heterogeneity, and *adj R^2^* explained the proportion of between-study variation due to heterogeneity [25,26]. An *I^2^ res* close to 100% and *adj R^2^* close to 0% further indicated no effects on heterogeneity. Pooled estimates were performed to sensitivity analysis which involved omitting one study at a time followed by recalculation to test for robustness of the summary effects [26]. To increase transparency, risk of bias ratings and meta-analyses were displayed together. Funnel plots were used to investigate the risk of publication bias [23]. Egger’s and Begg’s regression tests evaluated publication bias with quantitative analysis [27]. A *P*-value <0.05 was accepted as statistically significant.

## Results

### Study characteristics

**Figure 1:**
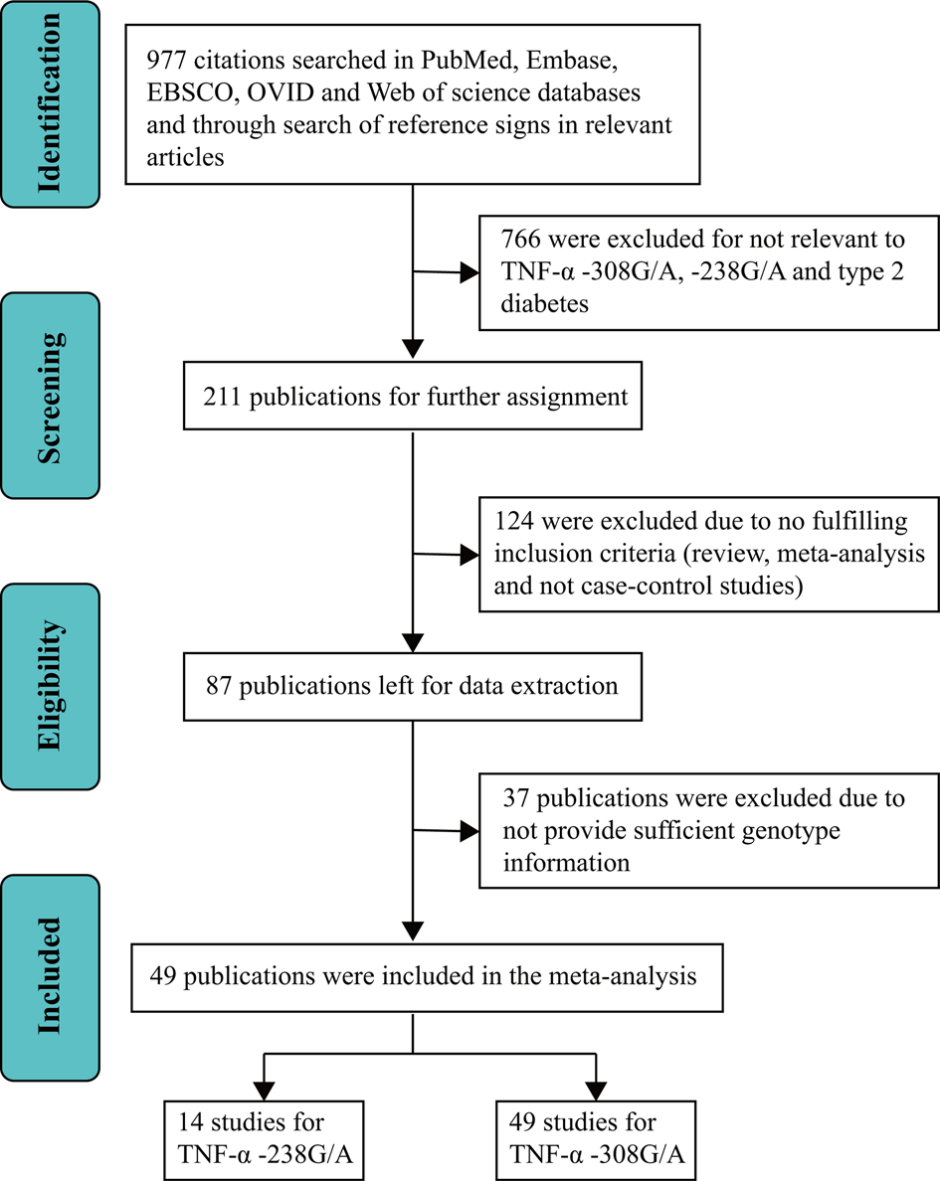
Study flow diagram

Based on the above search strategy, 977 publications were identified in the initial search. Approximately 766 articles were excluded after scanning titles and abstracts as being non- relevant to T2DM and *TNF-α* −308G/A and −238G/A. Through in-depth full-text analysis of the remaining 211 publications, 49 publications were used for the final meta-analysis (Figure 1). These 49 publications contained 16246 patients and 13973 controls and were included in the −308G/A analysis, of which 14 publications, with 4935 patients and 5260 controls, were included in the −238G/A analysis. According to NOS classifications, three points or lower indicated low quality, however no publications were of low quality. The main characteristics of selected publications are shown in Table 1.

**Table 1:** Characteristics of the included studies

Author	Year	Country	Ethnicity	Genotype in case				Genotype in control				*P* of HWE	NOS
*TNF-α* -308G/A				Total	GG (%)	GA (%)	AA (%)	Total	GG (%)	GA (%)	AA (%)		
Patel et al. [15]	2018	India	Asian	388	351(90.5%)	34 (8.8%)	3 (0.8%)	493	449 (91.1%)	42 (8.5%)	2 (0.4%)	0.348	6
Umapathy et al. [43]	2018	India	Asian	538	302 (56.1%)	142 (26.4%)	94 (17.5%)	218	167 (76.6%)	32 (14.7%)	19 (8.7%)	0.000^1^	4
Hemmed et al. [29]	2018	India	Asian	862	528 (61.3%)	283 (32.8%)	51 (5.9%)	464	356 (76.7%)	96 (20.7%)	12 (2.6%)	0.080	5
Fathy et al. [44]	2018	Kuwaiti	Caucasian	117	86 (73.5%)	28 (23.9%)	3 (2.6%)	42	41 (97.6%)	0 (0.0%)	1 (2.4%)	0.000^1^	6
Rodrigues et al. [45]	2017	Brazil	Caucasian	102	78 (76.5%)	23 (22.5%)	1 (1.0%)	62	47 (75.8%)	15 (24.2%)	0 (0.0%)	0.279	6
Mortazavi et al. [46]	2017	Iran	Caucasian	174	24 (13.8%)	101 (58.0%)	49 (28.2%)	185	68 (36.8%)	76 (41.1%)	41 (22.2%)	0.029^1^	5
Jamil et al. [47]	2017	India	Asian	100	88 (88.0%)	10 (10.0%)	2 (2.0%)	100	87 (87.0%)	12 (12.0%)	1 (1.0%)	0.433	7
Doody et al. [48]	2017	India	Asian	198	178 (89.9%)	18 (9.1%)	2 (1.0%)	204	189 (92.6%)	13 (6.4%)	2 (1.0%)	0.004^1^	7
Churnosov et al. [49]	2017	Russia	Caucasian	236	176 (74.6%)	53 (22.5%)	7 (3.0%)	303	242 (79.9%)	55 (18.2%)	6 (2.0%)	0.180	5
Sesti et al. [50]	2015	Britain	Caucasian	695	535 (73.7%)	176 (24.2%)	15 (2.1%)	170	129 (75.9%)	38 (22.4%)	3 (1.8%)	0.917	7
Golshani et al. [17]	2015	Iran	Caucasian	1038	737 (71.0%)	269 (25.9%)	32 (3.1%)	1023	871 (85.1%)	142 (13.9%)	10 (1.0%)	0.124	6
Dabhi et al. [51]	2015	India	Asian	214	185 (86.5%)	27 (12.6%)	2 (0.9%)	235	191 (81.3%)	44 (18.7%)	0 (0.0%)	0.885	4
Ghodsian et al. [52]	2015	Malaysia	Asian	88	73 (83.0%)	14 (15.9%)	1 (1.1%)	232	202 (87.1%)	29 (12.5%)	1 (0.4%)	0.970	6
Dhamodharan et al. [53]	2015	India	Asian	409	218 (53.3%)	117 (28.6%)	74 (18.1%)	106	77 (72.6%)	14 (13.2%)	15 (14.2%)	0.000^1^	5
Sikka et al. [54]	2014	India	Asian	462	405 (87.7%)	55 (11.9%)	2 (0.4%)	203	176 (86.7%)	27 (13.3%)	0 (0.0%)	0.310	7
Sharma et al. [55]	2014	India	Asian	51	45 (88.2%)	6 (11.8%)	0 (0.0%)	51	50 (98.0%)	1 (2.0%)	0 (0.0%)	0.944	5
Saxena et al. [56]	2013	India	Asian	213	173 (81.2%)	33 (15.5%)	7 (3.3%)	140	111 (79.3%)	25 (17.9%)	4 (2.9%)	0.095	6
Garcia-Elorriaga et al. [57]	2013	Mexico	Caucasian	51	41 (80.4%)	10 (19.6%)	0 (0.0%)	48	41 (85.4%)	2 (4.2%)	5 (10.4%)	0.000^1^	6
El Naggar et al. [16]	2013	Egypt	African	30	12 (40.0%)	12 (40.0%)	6 (20.0%)	15	9 (60.0%)	1 (6.7%)	0 (0.0%)	0.868	4
Mustapic et al. [58]	2012	Croatia	Caucasian	196	138 (70.4%)	55 (28.1%)	3 (15.0%)	456	336 (73.7%)	108 (23.7%)	12 (2.6%)	0.355	4
Perez-Luque et al. [30]	2012	Mexico	Caucasian	95	72 (75.8%)	23 (24.2%)	0 (0.0%)	87	82 (94.3%)	5 (5.7%)	0 (0.0%)	0.783	4
Wang et al. [59]	2012	China	Asian	100	74 (74.0%)	15 (15.0%)	11 (11.0%)	113	100 (88.5%)	12 (10.6%)	1 (0.9%)	0.359	5
Elsaid et al. [60]	2012	Egypt	African	69	10 (14.5%)	55 (79.7%)	4 (5.8%)	106	11 (10.4%)	94 (88.7%)	1 (0.9%)	0.000^1^	6
Liu et al. [32]	2011	China	Asian	112	67 (59.8%)	32 (28.6%)	13 (11.6%)	50	45 (90.0%)	5 (10.0%)	0 (0.0%)	0.710	5
Guzman-Flore et al. [61]	2011	Mexico	Caucasian	259	225 (86.9%)	31 (12.0%)	3 (1.2%)	645	573 (88.8%)	69 (10.7%)	3 (0.5%)	0.556	5
Mukhopadhyaya et al. [62]	2010	India	Asian	40	35 (87.5%)	3 (7.5%)	2 (5.0%)	40	37 (92.5%)	3 (7.5%)	0 (0.0%)	0.805	4
Boraska et al. [63]	2010	Britain	Caucasian	1454	938 (64.5%)	477 (32.8%)	39 (2.7%)	2504	1633 (65.2%)	774 (30.9%)	97 (3.9%)	0.659	6
Bouhaha et al. [64]	2010	Tunis	African	195	141 (72.3%)	51 (26.2%)	3 (1.5%)	299	204 (68.2%)	89 (29.8%)	6 (2.0%)	0.297	4
Liu et al. [13]	2008	China	Asian	245	222 (90.6%)	21 (8.6%)	2(0.8%)	122	109 (89.3%)	13 (10.7%)	0 (0.0%)	0.534	6
Lindholm et al. [65]	2008	Scandinavia	Caucasian	2927	1908 (65.2%)	906 (31.0%)	113(3.9%)	205	133 (64.9%)	66 (32.2%)	6 (2.9%)	0.520	4
Wang et al. [66]	2008	China	Asian	181	157 (86.7%)	23 (12.7%)	1 (0.6%)	82	67 (81.7%)	15 (18.3%)	0 (0.0%)	0.362	5
Kim et al. [34]	2006	Korea	Asian	198	174 (87.9%)	24 (12.1%)	0 (0.0%)	169	141 (83.4%)	28 (16.6%)	0 (0.0%)	0.240	4
Willer et al. [67]	2006	Finland	Caucasian	761	568 (74.6%)	184 (24.1%)	9 (1.2%)	617	469 (76.0%)	134 (21.7%)	14 (2.3%)	0.235	6
Santos et al. [68]	2006	Chile	Caucasian	30	27 (90.0%)	3 (10.0%)	0 (0.0%)	53	45 (84.9%)	8 (15.1%)	0 (0.0%)	0.552	4
Zeggini et al. [12]	2005	Britain	Caucasian	776	484 (62.4%)	260 (33.5%)	32 (4.1%)	1213	779 (64.2%)	391 (32.2%)	43 (3.5%)	0.480	6
Tsiavou et al. [69]	2004	Greece	Caucasian	32	29 (90.6%)	3 (9.4%)	0 (0.0%)	39	32 (82.1%)	7 (17.9%)	0 (0.0%)	0.538	4
Zouari et al. [70]	2004	Tunis	African	280	196 (70.0%)	64 (22.9%)	20 (7.1%)	274	170 (62.0%)	93 (33.9%)	11 (4.0%)	0.698	4
Shiau et al. [14]	2003	China	Asian	257	218 (84.8%)	35 (13.6%)	4 (1.6%)	187	168 (89.8%)	16 (8.6%)	3 (1.6%)	0.002^1^	5
Li et al. [71]	2003	Sweden	Caucasian	488	333 (68.24%)	141 (28.9%)	14 (2.9%)	284	189 (66.5%)	83 (29.2%)	12 (4.2%)	0.456	6
Heijmans et al. [72]	2002	Netherlands	Caucasian	79	51 (64.6%)	22 (27.8%)	6 (7.6%)	577	378 (65.5%)	189 (32.8%)	10 (1.7%)	0.012^1^	5
Furuta et al. [73]	2002	Japan	Asian	132	129 (97.7%)	3 (2.3%)	0 (0.0%)	142	139 (97.9%)	3(2.1%)	0(0.0%)	0.899	5
Rasmussen et al. [74]	2000	Danish	Caucasian	243	154 (63.4%)	79 (32.5%)	10 (4.1%)	325	214 (65.8%)	99 (30.5%)	12 (3.7%)	0.896	4
Kamizono et al. [75]	2000	Japan	Asian	213	209 (98.1%)	4 (1.9%)	0 (0.0%)	259	249 (96.1%)	10 (3.9%)	0 (0.0%)	0.751	4
Pandey et al. [76]	1999	Belgium	Caucasian	214	144 (67.3%)	61 (28.5%)	9 (4.2%)	200	145 (72.5%)	53 (26.5%)	2 (1.0%)	0.233	4
Hamann et al. [77]	1995	America	Caucasian	138	108 (78.3%)	27 (19.6%)	3 (2.2%)	57	46 (80.7%)	10 (17.5%)	1 (1.8%)	0.604	5
Kung et al. [78]	2010	China	Asian	23	0 (0.0%)	23 (100.0%)	0 (0.0%)	25	0 (0.0%)	25 (100.0%)	0 (0.0%)	0.000^1^	6
Ko et al. [79]	2003	China	Asian	339	284 (83.8%)	50 (14.7%)	5(1.5%)	202	171 (84.7%)	31 (15.3%)	0 (0.0%)	0.238	4
Morris et al. [80]	2003	Australia	Caucasian	91	53 (58.2%)	32 (35.2%)	6(6.6%)	189	126 (66.7%)	5 5(29.1%)	8 (4.2%)	0.427	4
Sobti et al. [81]	2012	India	Asian	113	5 (4.4%)	100 (88.5%)	8(7.1%)	158	26 (16.5%)	116 (73.4%)	16 (10.1%)	0.000^1^	5
*TNF-α* -238G/A				Total	GG (%)	GA (%)	AA (%)	Total	GG (%)	GA (%)	AA (%)		
Rasmussen et al. [82]	2000	Danish	Caucasian	236	205 (86.9%)	31 (13.1%)	0 (0.0%)	309	272 (88.0%)	35 (11.3%)	2 (0.6%)	0.459	4
Kim et al. [34]	2007	Korea	Asian	198	177 (89.4%)	21 (10.6%)	0 (0.0%)	169	152 (89.9%)	17 (10.1%)	0 (0.0%)	0.491	4
Sesti et al. [50]	2015	Britain	Caucasian	695	624 (89.8%)	66 (9.5%)	5 (0.7%)	169	147 (87.0%)	22 (13.0%)	0 (0.0%)	0.365	7
Santos et al. [68]	2006	Chile	Caucasian	30	28 (93.3%)	2 (6.7%)	0 (0.0%)	53	46 (86.8%)	7 (13.2%)	0 (0.0%)	0.607	4
Li et al. [71]	2003	Sweden	Caucasian	488	460 (94.3%)	27 (9.5%)	1 (0.2%)	284	265 (93.3%)	18 (6.3%)	1 (0.4%)	0.581	6
Dhamodharan et al. [53]	2015	India	Asian	133	100 (75.2%)	29 (21.8%)	4 (3.0%)	106	81 (76.4%)	23 (21.7%)	2 (1.9%)	0.806	5
Patel et al. [15]	2018	India	Asian	320	292 (91.3%)	27 (8.4%)	1 (0.3%)	295	257 (87.1%)	37 (12.5%)	1 (0.3%)	0.785	7
Fathy et al. [44]	2018	Kuwaiti	Caucasian	117	115 (98.3%)	2 (1.7%)	0 (0.0%)	42	41 (97.6%)	1 (2.4%)	0 (0.0%)	0.938	6
Boraska et al. [63]	2010	Britain	Caucasian	1504	1331 (88.5%)	170 (11.3%)	3 (0.2%)	2518	2224 (88.3%)	288 (11.4%)	6 (0.2%)	0.296	6
Zeggini et al. [12]	2005	Britain	Caucasian	560	470 (83.9%)	87 (15.5%)	3 (0.5%)	341	303 (88.9%)	37 (10.9%)	1 (0.3%)	0.908	6
Jamil et al. [47]	2017	India	Asian	98	85 (86.7%)	12 (12.2%)	1 (1.0%)	102	87 (85.3%)	13 (12.7%)	2 (2.0%)	0.094	7
Shiau et al. [14]	2003	China	Asian	257	218 (84.8%)	35 (13.6%)	4 (1.6%)	187	168 (89.8%)	16 (8.6%)	3 (1.6%)	0.002^1^	5
Guzman-Flore et al. [61]	2011	Mexico	Caucasian	259	220 (84.9%)	31 (12.0%)	8 (3.1%)	645	571 (88.5%)	71 (11.0%)	3 (0.5%)	0.622	5
Mukhopadhyaya et al. [83]	2010	India	Asian	40	35 (87.5%)	3 (7.5%)	2 (5.0%)	40	37 (92.5%)	3 (7.5%)	0 (0.0%)	0.805	4

1 Deviated from HWE.

### Overall population

**Figure 2:**
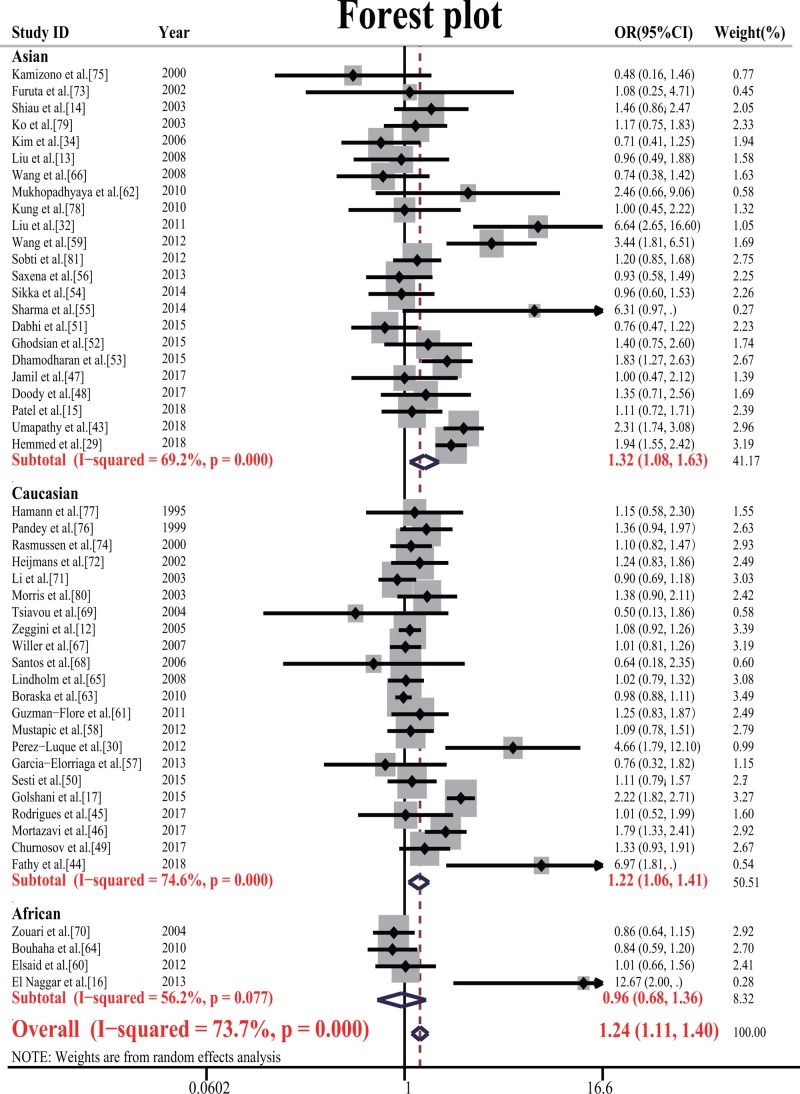
Forest plot of the association of *TNF-α* −308G/A and type 2 diabetes (A vs. G) in random-effects model Each square is proportional to the study-specific weight.

**Figure 3:**
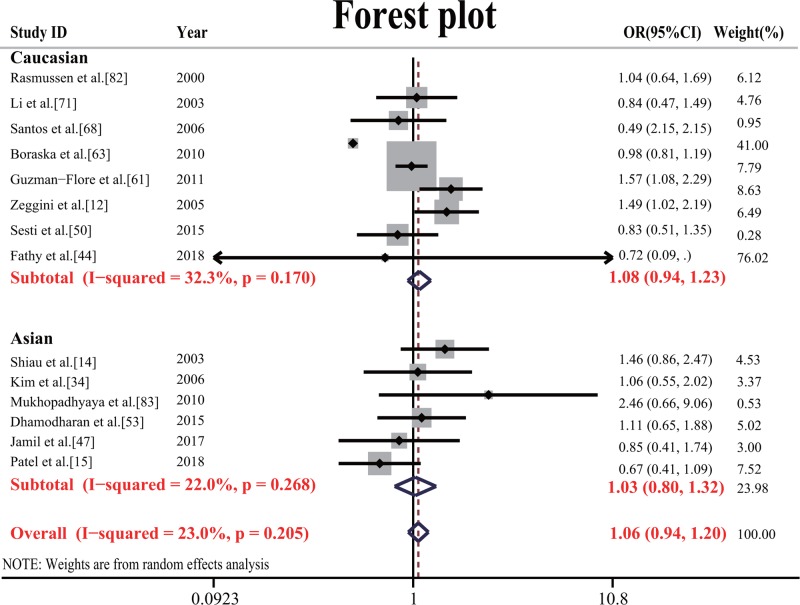
Forest plot of the association of *TNF-α* −238G/A and type 2 diabetes (A vs. G) in fixed-effects model Each square is proportional to the study-specific weight.

The meta-analysis showed a significant association between *TNF-α* −308G/A and T2DM risk in the allele model (OR = 1.239, 95% CI = 1.108–1.385, *P*=0.000); the dominant model (OR = 1.280, 95% CI = 1.116–1.469, *P*=0.000); the recessive model (OR = 1.446, 95% CI = 1.154–1.813, *P*=0.001); the overdominant model (OR = 1.181, 95% CI = 1.041–1.341, *P*=0.008); and the codominant model (OR = 1.691, 95% CI = 1.310–2.184, *P*=0.000). *TNF-α* −238G/A was not associated (*P*>0.05) with T2DM in all genetic models (Table 2). After Bonferroni correction, our results were also significantly associated. The forest plot of the −308G/A polymorphism is shown in Figure 2 and −238G/A is shown in Figure 3.

**Table 2:** Association between *TNF-α* -308G/A and -238G/A and type 2 diabetes

Genetic model	Ethnicity	*I^2^* (%)	*P* (heterogeneity)	OR (95% CI)	*P*-value	*P* for publication bias		Effects model
						Begg	Egger	
*TNF-α* -308G/A A vs G								
	Overall	73.7	0.000^‡^	1.239 (1.108–1.385)	0.000^‡^	0.268	0.000	Random
	Caucasian	74.6	0.000^‡^	1.224 (1.060–1.413)	0.006^†^	0.135	0.363	Random
	Asian	69.2	0.000^‡^	1.324 (1.078–1.626)	0.007^†^	0.809	0.249	Random
	African	56.2	0.077	0.960 (0.679–1.356)	0.815	0.174	0.015	Random
GA+AA vs GG								
	Overall	74.6	0.000^‡^	1.280 (1.116–1.469)	0.000^‡^	0.096	0.275	Random
	Caucasian	74.6	0.000^‡^	1.282 (1.085–1.514)	0.004^†^	0.069	0.376	Random
	Asian	71.7	0.000^‡^	1.367 (1.065–1.754)	0.014*	0.174	0.532	Random
	African	57.6	0.070	0.844 (0.522–1.363)	0.487	0.487	0.234	Random
AA vs GG+GA								
	Overall	38.3	0.008^†^	1.446 (1.154–1.813)	0.001^†^	0.207	0.125	Random
	Caucasian	51.3	0.005^†^	1.240 (0.908–1.692)	0.176	0.469	0.276	Random
	Asian	0.0	0.497	1.789 (1.357–2.357)	0.000^‡^	0.284	0.363	Random
	African	9.4	0.346	1.809 (0.890–3.677)	0.102	0.497	0.561	Random
GA vs GG+AA								
	Overall	67.8	0.000^‡^	1.181 (1.041–1.341)	0.008^†^	0.364	0.634	Random
	Caucasian	66.3	0.000^‡^	1.225 (1.050–1.423)	0.005^†^	0.243	0.594	Random
	Asian	63.7	0.000^‡^	1.230 (0.977–1.548)	0.079	0.846	0.619	Random
	African	50.5	0.109	0.707 (0.455–1.098)	0.123	0.174	0.452	Random
AA vs GG								
	Overall	47.4	0.001^‡^	1.691 (1.310–2.184)	0.000^‡^	0.285	0.068	Random
	Caucasian	62.8	0.000^‡^	1.399 (0.969–2.018)	0.073	0.506	0.244	Random
	Asian	0.0	0.842	2.368 (1.779–3.153)	0.000^‡^	0.365	0.157	Random
	African	11.6	0.335	1.605 (0.765–3.369)	0.211	1.000	0.942	Random
AA vs GA								
	Overall	31.8	0.029*	1.150 (0.918–1.441)	0.224	0.285	0.068	Random
	Caucasian	46.8	0.013*	1.031 (0.756–1.405)	0.847	0.506	0.244	Random
	Asian	0.0	0.533	1.138 (0.834–1.553)	0.414	0.365	0.157	Random
	African	0.0	0.414	2.230 (1.160–4.287)	0.016*	1.000	0.942	Random
*TNF-α* -238G/A A vs G								
	Overall	23.0	0.205	1.064 (0.944–1.200)	0.309	0.524	0.821	Fixed
	Caucasian	32.3	0.170	1.076 (0.938–1.234)	0.295	0.453	0.860	Fixed
	Asian	22.0	0.268	1.027 (0.802–1.316)	0.832	0.881	0.639	Fixed
GA+AA vs GG								
	Overall	8.3	0.362	1.045 (0.921–1.187)	0.936	0.396	0.947	Fixed
	Caucasian	15.8	0.306	1.056 (0.914–1.220)	0.459	0.293	0.801	Fixed
	Asian	13.5	0.328	1.011 (0.774–1.320)	0.492	0.881	0.719	Fixed
AA vs GG+GA								
	Overall	0.0	0.497	1.554 (0.896–2.692)	0.085	0.881	0.754	Fixed
	Caucasian	31.2	0.202	1.795 (0.888–4.533)	3.628	0.573	0.350	Fixed
	Asian	0.0	0.810	1.243 (0.516–2.977)	0.619	0.327	0.680	Fixed
GA vs GG+AA								
	Overall	0.0	0.462	1.021 (0.897–1.162)	0.758	0.396	0.908	Fixed
	Caucasian	4.1	0.398	1.029 (0.889–1.192)	0.698	0.453	0.689	Fixed
	Asian	8.4	0.363	0.990 (0.751–1.304)	0.943	0.652	0.813	Fixed
AA vs GG								
	Overall	0.00	0.496	1.569 (0.905–2.721)	0.078	0.881	0.748	Fixed
	Caucasian	31.6	0.198	1.807 (0.894–3.654)	0.064	0.348	0.414	Fixed
	Asian	0.0	0.811	1.262 (0.523–3.046)	0.596	0.142	0.356	Fixed
AA vs GA								
	Overall	0.0	0.533	1.429 (0.808–2.526)	0.178	0.881	0.748	Fixed
	Caucasian	24.3	0.252	1.688 (0.822–3.466)	0.117	0.348	0.414	Fixed
	Asian	0.0	0.778	1.079 (0.424–2.748)	0.852	0.142	0.356	Fixed

* *P*<0.05.

† *P*<0.01.

‡ *P*<0.001.

### Subgroup by ethnicity

To derive heterogeneity and assess the genetic background, we carried out a subgroup analysis, where the overall population was divided into three subgroups, namely Caucasian, Asian and African. The subgroup analysis showed significant associations between −308G/A and T2DM risk in the Caucasian population in the allele model (OR = 1.224, 95% CI = 1.060–1.413, *P*=0.006); the dominant model (OR = 1.282, 95% CI = 1.085–1.514, *P*=0.004); the overdominant model (OR = 1.225, 95% CI = 1.050–1.423, *P*=0.005), and also in Asian populations in the allele model (OR = 1.324, 95% CI = 1.078–1.626, *P*=0.007); the dominant model (OR = 1.367, 95% CI = 1.065–1.754, *P*=0.014); the recessive model (OR = 1.789, 95% CI = 1.357–2.357, *P*=0.000); the codominant model (OR = 2.368, 95% CI = 1.779–3.153, *P*=0.000) and no associations between −308G/A and T2DM risk in African populations (*P*>0.05). For −238G/A, it was not associated (*P*>0.05) with T2DM in the subgroup population (Table 2).

### Meta-regression and sensitivity analysis

**Figure 4:**
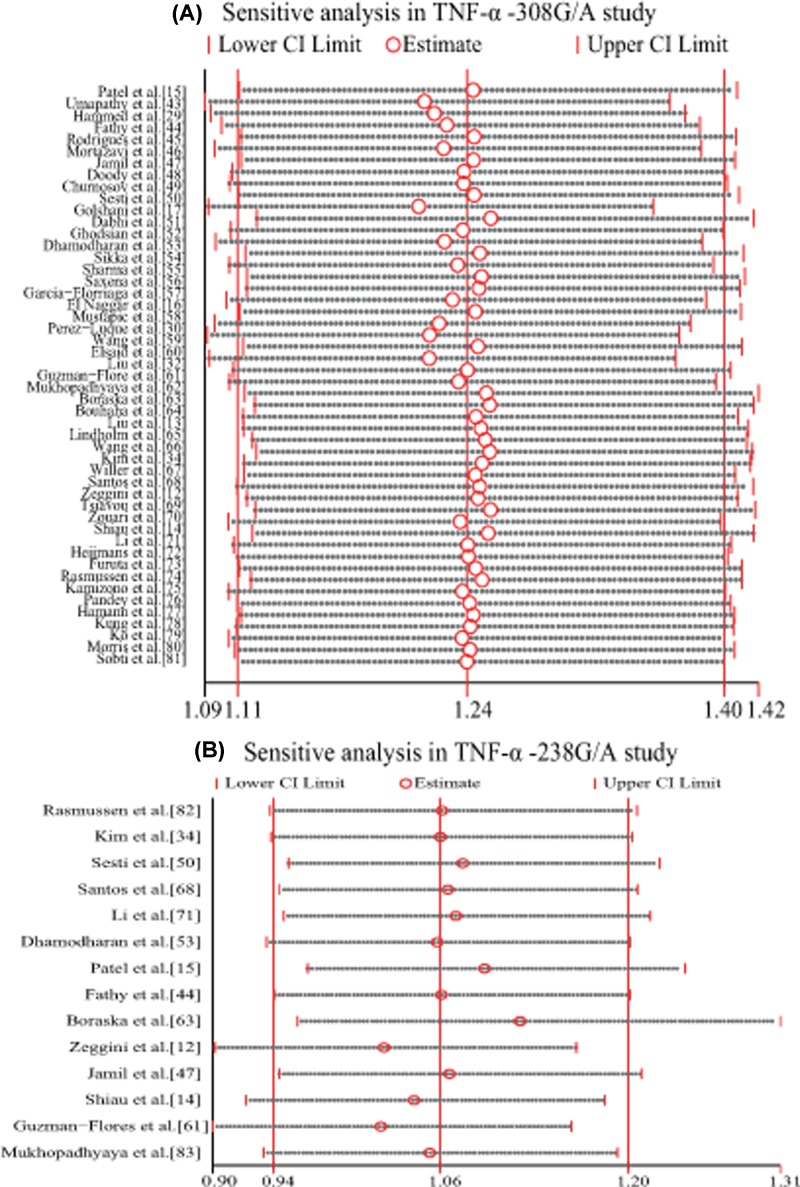
Sensitive analysis in *TNF-α* −308G/A study (**A**) and −238G/A study (**B**). There is a bias and asymmetry in *TNF-α−*308G/A study.

The following covariates were considered for meta-regression: publication year, sample size, ethnicity and HWE in controls. The −308G/A results revealed no influence on the publication year (*I^2^ res* = 91.89%, *adj R^2^* = 5.37%, *P*=0.084), sample size (*I^2^ res* = 94.31%, *adj R^2^*= 1.11%, *P*=0.215), HWE (*I^2^ res* = 92.83%, *adj R^2^*= −2.97%, *P*=0.882) and ethnicity, including Caucasian (*P*=0.106), Asian (*P*=0.127), using the 10000 times Monte Carlo permutation test. The −238G/A results revealed no influence from publication year (*P*=0.573), sample size (*P*=0.498) and ethnicity, including Caucasian (*P*=0.864) and Asian (*P*=0.735), using the 10000 times Monte Carlo permutation test. Sensitivity analysis revealed that some studies [17,28–32] have observed bias (Figure 4). But no significant changes in heterogeneity were observed after excluding these studies except study by Golshani et al. [17]. After its removal, the heterogeneity was greatly reduced in the Caucasian subgroup (from 74.6 to 47.4), but there was still a significant association between −308G/A and T2DM (OR = 1.148, 95% CI = 1.033–1.277, *P*=0.011).

### Publication bias

**Figure 5:**
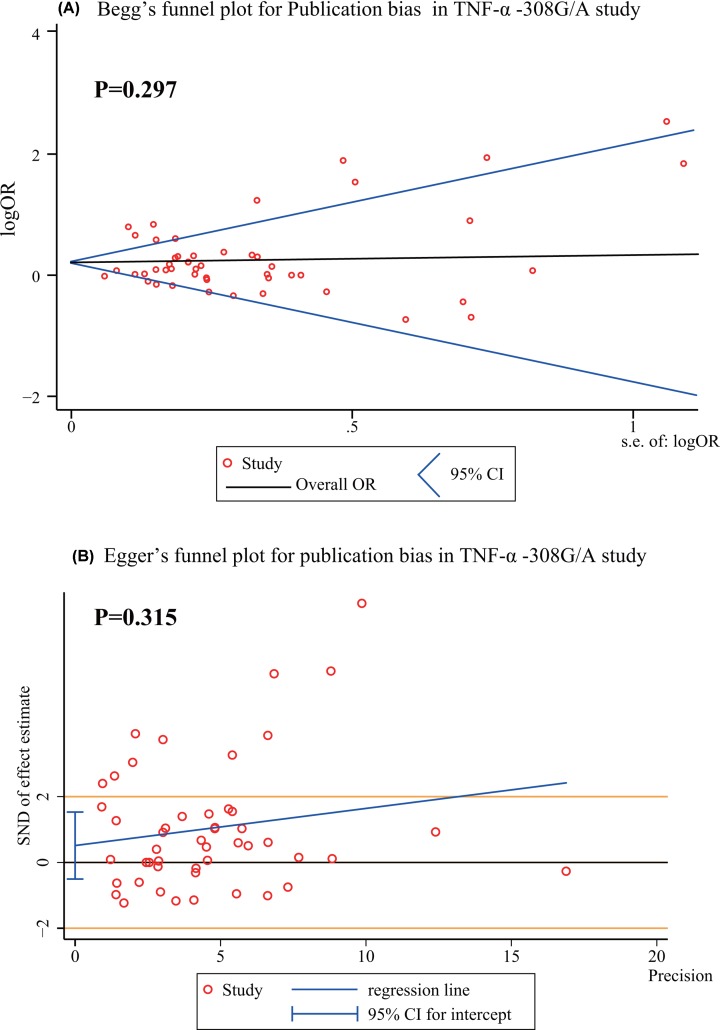
Publication bias of Begg’s test (**A**) and Egger’s test (**B**) in *TNF-α −*308G/A study Begg’s funnel plot shows centered at the fixed-effect summary OR, visual inspection of the funnel plot is roughly symmetrical and indicates that there is no bias (*P*>0.05). Egger’s funnel plot with fitted regression line, intercept represents the degree of asymmetry, close to zero, the smaller the bias. The Egger’s test indicates that there are no small-study effects (intercept = 0.514, 95% CI = −1.504–1.532) and bias (*P*>0.05).

**Figure 6:**
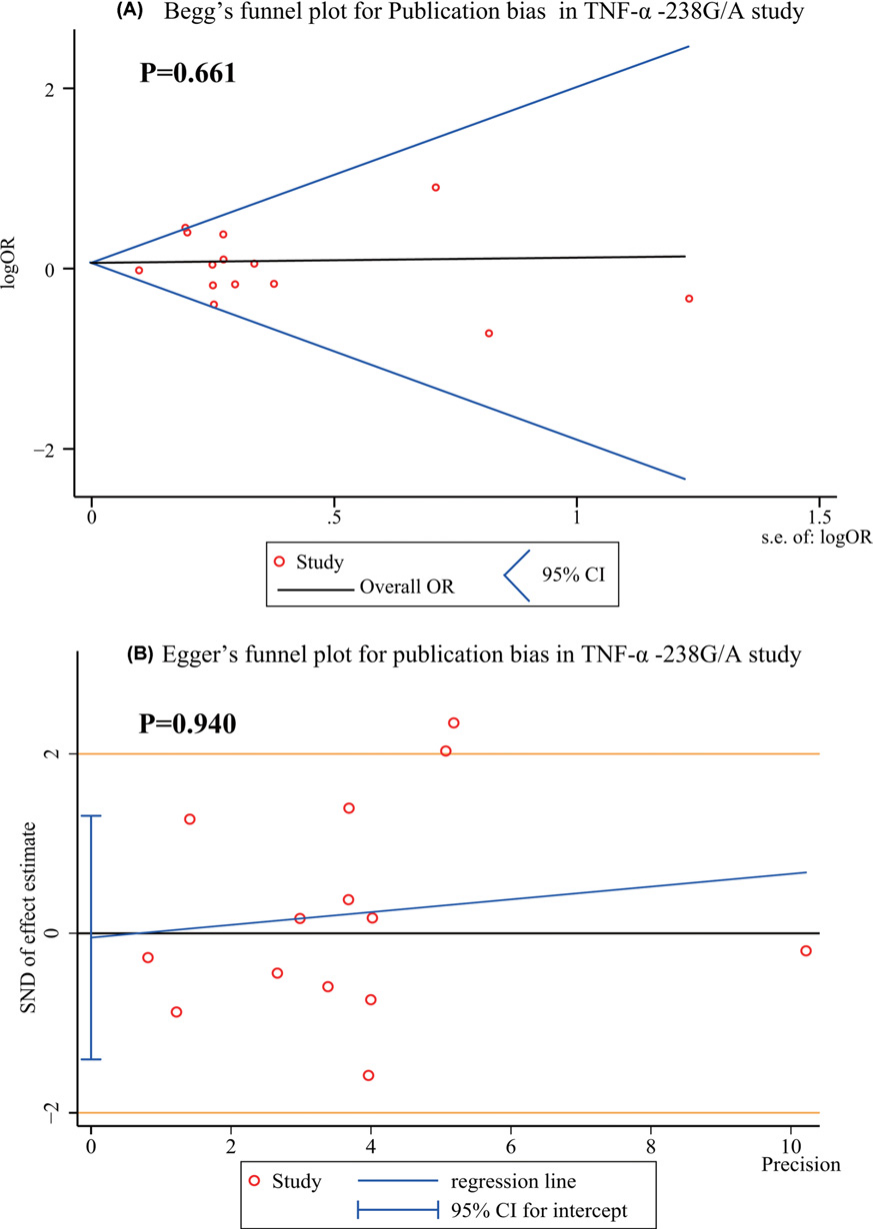
Publication bias of Begg’s test (**A**) and Egger’s test (**B**) in *TNF-α −*238G/A study Begg’s funnel plot shows centered at the fixed-effect summary OR, visual inspection of the funnel plot is roughly symmetrical and indicates that there is no bias (*P*>0.05). Egger’s funnel plot with fitted regression line, intercept represents the degree of asymmetry, close to zero, the smaller the bias. The Egger’s test indicates that there are no small-study effects (intercept = −0.048, 95% CI = −1.405–1.309) and bias (*P*>0.05).

Publication bias data for *TNF-α* −308G/A and −238G/A, in all genetic models are shown in Table 2. The continuity corrected results showed no existing publication bias (*P*>0.05). The Begg’s and Egger’s tests showed no existing publication bias in the overall population for all genetic models (Table 2). There are no bias and asymmetry found in Begg’s and Egger’s funnel plots (Figures 5 and 6).

## Discussion

T2DM is a complex disease where environmental and genetic factors interact. Family-based studies have found that T2DM has a strong genetic component [33] with several candidate genes identified [1]. Among these candidate genes, the *TNF-α* −308G/A and −238G/A polymorphisms have been widely studied. Although numerous studies have focused on these associations, their conclusions have been controversial [13,17,34,35]. A previous meta-analysis by Feng et al. [36], did not find any significant associations between the *TNF-α* −308 G/A polymorphism and T2DM risk in Caucasian and Asian populations. In contrast, a more recent meta-analysis by Zhao et al. [37], suggested that the *TNF-α* −308A variant increased by approximately 21% in T2DM incidence. Similarly, the results of two meta-analyses, of small sample sizes, showed that *TNF-α* −238G/A was not associated with T2DM [38,39]. Moreover, some meta-analyses were limited to specific countries and regions [40–42]. Therefore, we performed a comprehensive large-scale meta-analysis to investigate these associations.

For this meta-analysis, in order to derive reliable results, we added 12 new studies, performed quality score assessments and added multiple genetic models. Compared with previous meta-analyses [36,37], we demonstrate that *TNF-α* −308G/A is a risk factor for T2DM, not only in Asian but also in Caucasian populations. Additionally, we found that *TNF-α* −238G/A is not associated with T2DM in overall and subgroup populations. These observations illustrate the necessity for more comprehensive analyses and multiple genetic models.

To prevent possible interference from heterogeneity to our results, we sought to explain the source of heterogeneity and eliminate it. First, subgroup analysis of ethnicity and genetic models reduced between-study heterogeneity. We found that heterogeneity was reduced, but there was still high heterogeneity. Next, our meta-regression analysis attempted to reveal these heterogeneous sources. These results showed that publication year, sample size, ethnicity (Caucasian, Asian, African) and HWE were not the sources of between-study heterogeneity (*P*>0.05). Finally, we performed sensitivity analysis to explore the impact of a single study; our results revealed that the study by Golshani et al. [17] may have been the major contributor to this heterogeneity.

The advantages of this meta-analysis are that it expands to large-scale studies. While strictly complying with the inclusion criteria, we updated 12 studies not included in previous meta-analysis, our results are more comprehensive. To guarantee the quality of the meta-analysis, NOS and HWE analyses were conducted to assess the quality of included studies to avoid potential influences and increase the strength of the results. A strict search strategy of literature inclusion and data extraction was performed by two investigators according to inclusion and exclusion criteria. Furthermore, sensitivity analysis and meta-regression were also performed to increase the robustness of our conclusions. Subgroup analysis by ethnicity and the source of the control population were used to explain the effect of genetic background and study design.

There were some limitations to this meta-analysis. First, only studies in English were included, studies published in other languages were excluded. Second, because we excluded literature without original data, some studies were excluded. Third, other potential interactions including environmental factors, environment–gene interactions and gene–gene interactions. Additionally, some potential covariates (e.g. age, sex) were not included due to insufficient information from selected publications.

In conclusion, our meta-analysis identified that *TNF-α* −308G/A were associated with T2DM susceptibility. Additionally, we found that *TNF-α* −238G/A is not associated with T2DM in overall and subgroup populations. In the future, the influences of genetic loci, combined with environmental factors, may provide important treatment therapies for T2DM, therefore, well-conceived studies are warranted to confirm the important data presented here.

## Abbreviations

GWAS: genome-wide association scans
HWE: Hardy–Weinberg equilibrium
NOS: Newcastle–Ottawa Quality Assessment Scale
OR: odds ratio
TNF-α: tumor necrosis factor-α
T2DM: type 2 diabetes mellitus
95% CI: 95% confidence interval
